# Recombinant Detoxified Holotoxin as a Potent Candidate Vaccine Against Botulism

**DOI:** 10.3390/vaccines13030243

**Published:** 2025-02-26

**Authors:** Zhixin Meng, Chunlin Cheng, Guoqing Xiong, Jiazhen Cui, Yuzhong Feng, Zhili Chen, Yuanyuan Lu, Xuan Huang, Qi Yang, Qi Xin, Xin Ge

**Affiliations:** 1 Key Laboratory of Microbial Diversity Research and Application of Hebei Province, Department of Microbial and Biochemical Pharmaceutics, College of Life Science, Hebei University, 180 Wusi East Road Street, Baoding 071002, China; mengzhixin68@163.com (Z.M.);; 2 Academy of Military Medical Sciences, Beijing 100071, China; 3 Lanzhou University of Information Science and Technology, Lanzhou 730300, China

**Keywords:** botulinum neurotoxin, botulism, detoxified holotoxin, toxicity, immunogenicity

## Abstract

Vaccination may represent a suitable strategy for preventing botulism. The recombinant expression of toxin functional domains can induce effective immune responses against botulism. This study aims to develop a safe and effective recombinant detoxified full-length BoNTA vaccine. In this study, we engineered and mutated the toxin activity-related sites on the basis of the full-length BoNTA protein and constructed three detoxified full-length toxin mutants. They were recombinant expressed and purified in *Escherichia coli*, and the BoNTA/M4 mutant was determined to have the highest safety, with a murine lethal dose of 50% (MLD_50_). The M4 protein was used as the antigen for three immunizations, and the serum titers, neutralizing activity, and BoNTA protective effects of immunized mice were evaluated. The results show that, in comparison to the receptor-binding domain BoNTA/Hc protein, the full-length detoxified mutant M4 protein exhibited superior immunogenicity and could induce higher levels of specific antibodies, and the resulting immune serum could effectively protect mice against higher doses of BoNTA challenge. This study laid the foundation for research on a novel recombinant detoxified full-length botulinum toxin vaccine.

## 1. Introduction

Botulism is a severe neurological paralysis disease characterized by descending flaccid paralysis. In the early stages, patients often exhibit initial symptoms of cranial nerve paralysis, followed by progressively worsening symptoms such as limb weakness, difficulty breathing, and, in severe cases, respiratory failure, complete muscle paralysis, and even death. The causative agent of botulism is the botulinum neurotoxins (BoNTs) secreted by *Clostridium botulinum* [1]. Due to its high toxicity and relatively easy preparation, BoNTs is classified as a “Category A bioterrorism agent” by the Centers for Disease Control and Prevention (CDC) in the United States [1]. Despite numerous preventive measures, foodborne and iatrogenic botulism poisoning incidents still occur occasionally [2].

BoNTs have at least seven different serotypes ranging from A to G, among which serotypes A, B, C, E, and F are capable of causing human poisoning, with serotype A being the primary serotype responsible for human botulism [3,4]. The newly translated BoNT/A is a single-chain protein with a molecular weight of approximately 150 kDa, which is proteolytically hydrolyzed into a 50 kDa light chain (LC) and a 100 kDa heavy chain (HC); these are tightly linked by a pair of disulfide bonds. The LC functions as a zinc-dependent metalloprotease, while HC is composed of an N-terminal translocation domain (H_N_) and a receptor-binding domain (Hc) [5]. The poisoning process of BoNTA can be divided into three main steps. Initially, the toxin travels through the bloodstream to reach the peripheral nervous system, where the receptor-binding domain of the heavy chain binds to specific receptors on the presynaptic membrane of motor neurons, forming endocytic vesicles and causing BoNTA to be internalized by the cells [6]. Subsequently, the H_N_ domain of the heavy chain forms a channel on the endocytic vesicle membrane, allowing for the LC to be transported into the cytoplasm of neurons [7]. Once LC enters the cytoplasm, it refolds into an active conformation and specifically cleaves the SNARE protein, blocking the release of the neurotransmitter acetylcholine and causing flaccid muscular paralysis [8]. When this paralysis affects the respiratory muscles, it can lead to respiratory failure and death [9].

Vaccination may represent a suitable strategy for preventing botulism [10,11]. The currently marketed vaccines are an equine antitoxin, but they suffer from issues such as limited availability, adverse reactions, decreased effectiveness, and weakened immune responses [12,13]. A pentavalent (ABCDE) botulinum toxoid vaccine (PBT) was developed in the United States, intended for high-risk laboratory workers and military personnel, but it was not approved by the Food and Drug Administration (FDA) due to the risk of allergic reactions and other side effects and is currently discontinued [14,15]. Research has found that recombinant expressed and purified receptor-binding domains (Hc) provide immune protection against BoNT challenge, and LC-HN domains also contain neutralizing epitopes that can effectively protect animals against BoNT challenge [16,17,18]. The use of a single structural domain of BoNT to some extent limits the immune protection effect. In contrast, the full-length detoxified protein of BoNTA contains all structural domains, offering more potential neutralizing epitopes [19]. Moreover, the full-length detoxified protein is structurally closer to natural BoNT than BoNT/Hc expressed alone [19].

Webb et al. successfully constructed a catalytic inactivated BoNT/A1 whole protein (ciBoNT/A1 HP) by mutating three key amino acid residues (H223A, E224A, H227A) on the BoNT/A1 light chain. This protein exhibited strong protective properties after immunization in mice and effectively resisted attacks from different subtypes of toxins BoNT/A1, A2, A3 [16]. Barbie et al. further studied three amino acid mutations (E224A, R363A, Y366F) in the light chain of recombinant full-length BoNT/A1, constructing M-BoNT/A1 to eliminate catalytic activity. [20]. Based on these findings, we identified multiple amino acid sites closely related to toxic functions on toxin LC that were reported in the literature, including H223, H227, E262 (which chelate with Zn^2^⁺), E224 (which interacts with water molecules), and R363, Y366 (which affect the stability of the transition state) [21,22,23,24,25,26,27]. After analyzing the crystal structure of the complex between BoNTA and receptor SNAP25, combinatorial mutations were carried out at the aforementioned sites. Three full-length mutant proteins, BoNTA/M2 (E224A/E262A), BoNTA/M4 (E224A/E262A/R363A/Y366F), and BoNTA/M6 (H223A/E224A/H227A/E262A/R363A/Y366F), were designed for recombinant expression, purification, and a toxicity evaluation. The highest safety mutant protein was selected to immunize mice, and its serum titers, neutralizing titers, and antiviral protection effects were evaluated and compared with BoNTA/Hc antigens.

## 2. Materials and Methods

### 2.1. Animals

ICR mice of 18–20 g and 18–20 g male KM mice were purchased from Sibeifu Biotechnology (Beijing, China) and housed in an SPF-grade environment in the animal room of the Beijing Academy of Military Medical Sciences in China. In accordance with the relevant institutional regulations on animal care, the mice were provided with sufficient drinking water and standardized mouse feed.

### 2.2. Construction of Mutant Expression Plasmids

The prokaryotic expression vector pTIG-BoNTA was stored in our laboratory [28]. The primers used in this study are shown in Table 1. PCR amplification was carried out using the E224A-F and E224A-R primer pairs with plasmid pTIG-BoNTA as the template. The PCR reaction conditions included an initial denaturation at 98 °C for 30 s, followed by 30 cycles of denaturation at 98 °C for 10 s, annealing at 55 °C for 30 s, and extension at 72 °C for 5 min. A final extension was performed at 72 °C for 10 min. The template plasmid was then digested with DpnI enzyme, inactivated at 80 °C for 20 min, and transformed into Escherichia coli DH5α competent cells. Positive transformants were selected for sequencing, confirming the correct sequencing of the mutant pTIG-BoNTA-E224A. Based on this plasmid, the pTIG-BoNTA-M2 (E224A/E262A) plasmid was constructed by adding the E262A-F and E262A-R primer pairs and repeating the PCR, digestion, inactivation, and transformation steps. Similarly, the pTIG-BoNTA/M4 and pTIG-BoNTA/M6 plasmids were constructed in sequence.

### 2.3. Recombinant Expression, Purification, and Characterization of Mutant Proteins

To transform the pTIG-BoNTA/M2, pTIG-BoNTA/M4, and pTIG-BoNTA/M6 plasmids into competent *Escherichia coli* BL21 (DE3) cells (Beijing TransGen Biotech Co., Ltd., Beijing, China), the recombinant strain was cultured in LB medium (Beijing CoolLab Technology Co, Ltd., Beijing, China) with Ampicillin (100 µg/mL) at 37 °C and 220 rpm until reaching OD600 ≈ 0.6. Induction was initiated by adding IPTG (Beijing Solebo Technology Co., Ltd., Beijing, China) to a final concentration of 1 mM, and the cells were then incubated at 20 °C and 220 rpm for 24 h. After induction, the bacterial cells were harvested via centrifugation at 8000 rpm and 4 °C for 20 min, resuspended in a buffer solution (20 mM Tris HCl, pH 7.4), and sonicated on ice. The supernatant was obtained via centrifugation at 12,000 rpm and 4 °C for 30 min.

The process of protein purification involves the following steps: (1) ammonium sulfate precipitation: ammonium sulfate is added to the supernatant after centrifugation to a saturation of 30%, followed by overnight incubation at 4 °C and centrifugation at 12,000 rpm to collect the supernatant; (2) hydrophobic chromatography: the supernatant is loaded onto with a Butyl HP column (Cytiva, MD, USA) and eluted with a gradient elution buffer (20 mM Tris-HCl, pH 7.4); (3) cation exchange chromatography: the protein-containing solution from the Butyl hydrophobic chromatography is diluted 20 times with starting buffer (20 mM Tris-HCl, pH 7.4) and loaded onto an SP HP column (Cytiva, MD, USA), followed by gradient elution with an elution buffer (20 mM Tris-HCl, 1 M NaCl, pH 7.4) to collect the target protein. Finally, the purity of the target protein was confirmed using SDS-PAGE and HPLC. The specificity of the target protein was identified using Western blot with the human monoclonal antibody ML01 against BoNTA/Hc.

### 2.4. Determination of Mutant Proteins Toxicity

Based on the results of the preliminary experiment, 18–20 g ICR mice were randomly divided into 15 groups, with 4 mice per group [29]. Groups 1–5 were injected with BoNTA/M2 protein at doses of 10 μg, 5 μg, 2 μg, 1 μg, and 500 ng, respectively. Groups 6–10 were injected with BoNTA/M4 protein at doses of 1 μg, 500 ng, 200 ng, 100 ng, and 50 ng, respectively. Groups 11–15 were injected with BoNTA/M6 protein at doses of 10 μg, 5 μg, 2 μg, 1 μg, and 500 ng, respectively, with dilution in PBS buffer at different concentrations. Each mouse was injected intraperitoneally with 500 μL of the dilution, and their survival status was monitored for 7 days. The LD_50_ values for the three proteins were calculated using IBM SPSS Statistics 26 software.

### 2.5. Mutant Protein Immunization

KM male mice of 18–20 g were randomly divided into three groups—the BoNTA/M4 group, positive control BoNTA/Hc group, and negative control PBS group—with 30 mice in each group [29]. BoNTA/M4, BoNTA/Hc, and 0.2% (*w*/*w*) aluminum hydroxide gel adjuvant (Beijing Solebo Technology Co., Ltd., Beijing, China) were mixed and emulsified in a 1:1 volume ratio to prepare the vaccine. The negative control PBS was also mixed with 0.2% (*w*/*w*) aluminum hydroxide gel adjuvant in the same way, at a volume ratio of 1:1. The immune doses of BoNTA/M4 and BoNTA/Hc proteins were 1.5 μg/mouse and 0.5 μg/mouse (equimolar amounts), immunized via intraperitoneal injection. After the first immunization, a booster immunization was offered every 2 weeks for a total of three immunizations. Mouse blood was collected 7 days after the second and third immunizations, centrifuged at 4000 rpm for 10 min to obtain serum, and stored at −80 °C for future use.

### 2.6. Serum Titration and Antibody Subtype Determination

ELISA was used to detect antigen-specific antibodies in mouse serum. Briefly, 100 ng of BoNTA/Hc antigen was coated onto the ELISA plates overnight at 4 °C. After blocking with TBST buffer containing 2% bovine serum albumin (BSA), 100 µL of diluted serum samples was added to the plates and incubated for 1 h. Anti-mouse IgG-F(ab)2-horseradish peroxidase (HRP) secondary antibody (Biotron Immunotechnology Co, Ltd., Suzhou, China) was diluted at a ratio of 1:5000 and incubated at room temperature for 1 h. For the analysis of antigen-specific IgG1, IgG2a, IgG2b, IgG3, and IgM in mouse serum, specific secondary antibodies (Biotron Immunotechnology Co., Ltd., Suzhou, China) were used for each subclass. The plates were washed five times, and a color reaction was developed through the addition of a tetramethylbenzidine (TMB) substrate, which was then terminated with 2 M H_2_SO_4_. The optical density at 450 nm (OD450 nm) was read using a BioTek microplate reader (Gujarat, India). The antibody titer was defined as the highest serum dilution ratio that resulted in an OD450 nm value 2.1 times greater than the background value of the serum samples from unimmunized mice.

### 2.7. Determination of Serum Neutralization Potency

The mouse serum collected after three immunizations (1 μL, 0.1 μL, and 0.01 μL) was mixed with BoNT/A (100 LD_50_), then added to PBS to create a total volume of 500 μL. The mixture was then incubated at 37 °C for 1 h. Male KM mice of 18–20 g were randomly divided into 9 groups, with 10 mice per group. A total of 500 μL of the above mixture was injected intraperitoneally into each mouse, and the survival status of the mice was recorded after 7 days.

### 2.8. Efficacy Studies

Fourteen days after the third immunization of the mice, the mice in the BoNTA/M4 group, positive control BoNTA/Hc group, and negative control PBS group were divided into six subgroups, with 10 mice per subgroup. They were then challenged with doses of 10^4^ LD_50_, 10^5^ LD_50_, and 10^6^ LD_50_ of BoNTA, and the survival of the mice was recorded for 7 days.

### 2.9. Data Statistical Analysis

A statistical analysis of the data was carried out using GraphPad software version 8.0.2, with variance analysis and chi-square tests used for comparison. Significance levels were set at *p* < 0.05 for significant differences, *p* < 0.01 for highly significant differences, and *p* < 0.0001 for extremely significant differences.

## 3. Results

### 3.1. Purification and Characterization of Mutant Proteins

**Figure 1:**
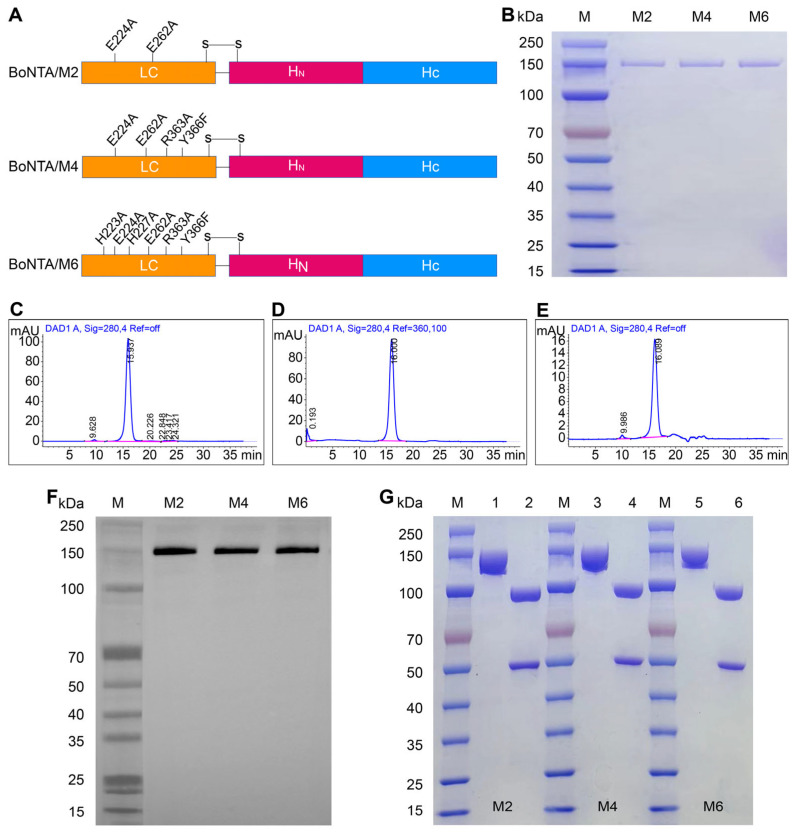
Purification and characterization of recombinant BoNTA/M2, BoNTA/M4, and BoNTA/M6. (**A**) Schematic diagrams of BoNTA/M2, BoNTA/M4, and BoNTA/M6; (**B**) SDS-PAGE analysis of purified BoNTA/M2, BoNTA/M4, and BoNTA/M6 proteins (10 μg protein/lane). 1: BoNTA/M2; 2: BoNTA/M4; 3: BoNTA/M6; (**C**) HPLC analysis of BoNTA/M2 protein; (**D**) HPLC analysis of BoNTA/M4 protein; (**E**) HPLC analysis of BoNTA/M6 protein; (**F**) WB analysis of BoNTA/M2, BoNTA/M4, and BoNTA/M6, with a human monoclonal antibody ML01 against BoNTA/Hc; (**G**) recombinant protein, analyzed using non-reduced and reduced SDS-PAGE electrophoresis (20 μg protein/lane). 1, 3, 5: non-reduced; 2, 4, 6: reduced.

The mutation sites of BoNTA/M2, BoNTA/M4, and BoNTA/M6 are illustrated in Figure 1A. We successfully purified the mutant proteins BoNTA/M2, BoNTA/M4, and BoNTA/M6 from Escherichia coli lysate using a combination of 30% saturated ammonium sulfate precipitation, butyl column hydrophobic chromatography, and SP column ion exchange chromatography. The SDS-PAGE results demonstrated that the purified mutant proteins were visually free of other impurities (Figure 1B). A high-performance liquid chromatography (HPLC) analysis revealed that the purity of the target proteins was as high as 96.69% (Figure 1C), 93.92% (Figure 1D), and 97.42% (Figure 1E), all of which were above 90% and met the purity standards for biological products. Western blot analysis confirmed that the purified recombinant BoNTA/M2, BoNTA/M4, and BoNTA/M6 proteins were able to specifically bind to the anti-BoNTA human monoclonal antibody ML01 (Figure 1F). These experimental results indicate that the size and structure of the purified mutant proteins are correct, and their purity meets the requirements for subsequent experiments. To verify the double-strand cleavage of the recombinant BoNTA/M2, BoNTA/M4, and BoNTA/M6 proteins, reduced and non-reduced SDS-PAGE electrophoresis analyses were conducted. Under non-reducing conditions, the recombinant proteins appeared as single chains with a molecular weight of approximately 150 kDa. Under reducing conditions, the mutant proteins were completely cleaved into two bands of 50 kDa and 100 kDa, corresponding to the light chain (LC) and heavy chain (HC), respectively (Figure 1G). This finding indicates that all three mutant proteins were completely cleaved into double chains linked by disulfide bonds.

### 3.2. LD_50_ Determination of Mutant Proteins

We determined the median lethal dose (LD_50_) of recombinant BoNTA/M2, BoNTA/M4, and BoNTA/M6 proteins in mice through lethal experiments. As shown in Table 2, the LD_50_ of recombinant BoNTA/M2, BoNTA/M4, and BoNTA/M6 protein antigens are 136.77 ng, 5000 ng, and 674 ng, respectively. Among them, the LD_50_ value of recombinant BoNTA/M4 is the highest, at 1 million times (or 10^6^ times) that of natural BoNTA (0.005 ng), indicating that the toxicity of the recombinant BoNTA/M4 was reduced by 1 million times compared to that of natural BoNTA. We chose the BoNTA/M4 with the highest safety levels for subsequent mouse immunization and evaluation.

### 3.3. Determination of Humoral Immune Response After Immunization with BoNTA/M4 Antigens

**Figure 2:**
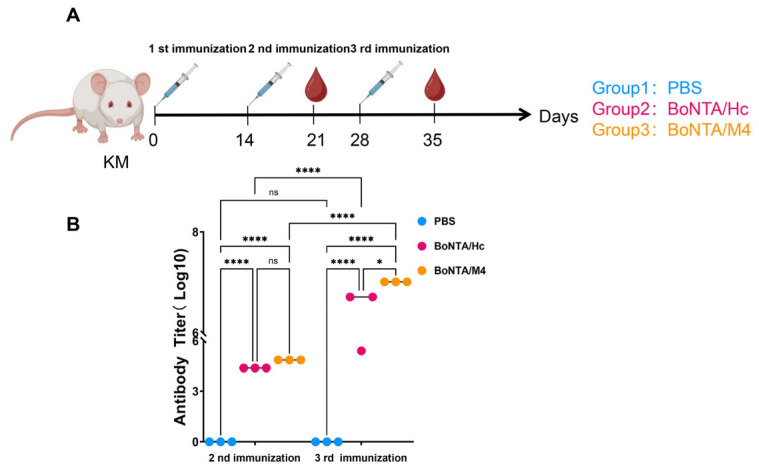
Antigen-specific antibody titers in mouse serum after secondary and tertiary immunization with BoNTA/M4, BoNT/A/Hc, and PBS. Vaccines were prepared by mixing BoNTA/M4, BoNT/AHc, or PBS with an aluminum hydroxide gel adjuvant and administered to mice via intraperitoneal injection. Serum samples were collected 7 days after the second and third immunizations. Antigen-specific antibody titers were evaluated by ELISA. (**A**) Immunization and blood sampling. (**B**) Antibody titers in mouse serum after secondary and tertiary immunization with BoNTA/M4, BoNT/AH_C_, or PBS. Data are presented as mean ± SEM (n = 3). * *p* < 0.05, **** *p* < 0.0001, ns, not significant (*p* > 0.05).

After three immunizations with BoNTA/M4 antigen, mouse serum antibody titers were measured using ELISA. KM mice were immunized with the BoNTA/M4 protein every two weeks, and blood samples were collected 7 days after the second and third immunizations for an ELISA analysis of serum antibody levels (Figure 2A). Compared to the negative control PBS group, the serum antibody levels of mice in the BoNTA/M4 sample group and BoNTA/Hc positive control group were significantly increased (*p* < 0.0001). The serum antibody titers of the BoNTA/M4 and positive control BoNTA/Hc mice after the second immunization were 1:72,900 and 1:24,300, respectively. After the third immunization, the antibody titers reached 1:2,028,000 and 1:5,120,000, respectively. The antibody levels of mice in the sample group and positive control group were significantly enhanced after the third immunization compared to the second immunization (*p* < 0.0001), with the BoNTA/M4 group mice showing significantly higher antibody levels than the BoNTA/Hc group (*p* < 0.05) (Figure 2B).

### 3.4. Types of Immune Response Immunized with BoNTA/M4 Antigen

**Figure 3:**
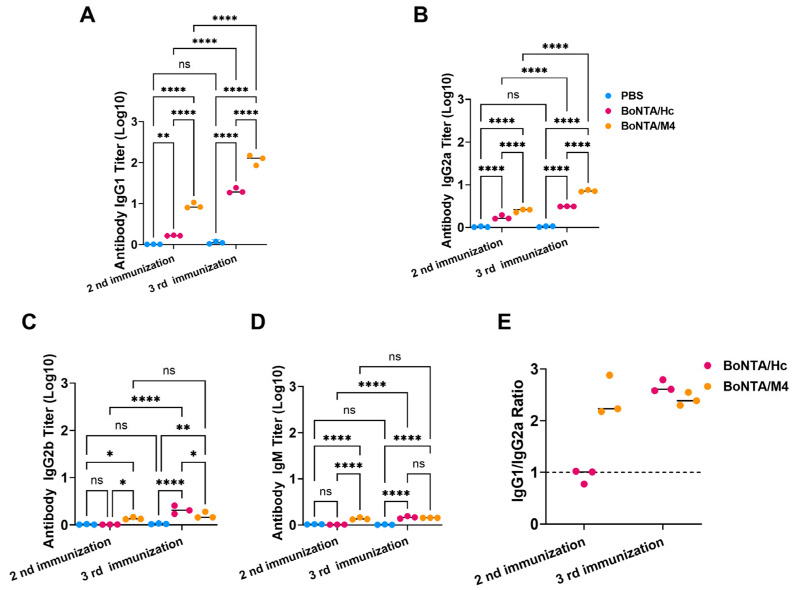
Subtype analysis of mouse serum antibodies after secondary and tertiary immunization with BoNTA/M4, BoNTA/Hc, and PBS. BoNTA/M4, BoNTA/Hc, and PBS were mixed with aluminum hydroxide gel adjuvant to prepare vaccines for the intraperitoneal immunization of mice. Serum samples were collected from mice 7 days after the second and third immunizations. Mouse serum antibody subtypes were evaluated using ELISA. (**A**) Titers of IgG1 antibodies in mouse serum after secondary and tertiary immunization with BoNTA/M4, BoNTA/HC, and PBS. (**B**) Titers of IgG2a antibodies in mouse serum after secondary and tertiary immunization with BoNTA/M4, BoNTA/Hc, and PBS. (**C**) Titers of IgG2b antibodies in mouse serum after secondary and tertiary immunization with BoNTA/M4, BoNT/AHc, and PBS. (**D**) Titers of IgM antibodies in mouse serum after secondary and tertiary immunization with BoNTA/M4, BoNTA/Hc, and PBS. (**E**) The IgG1/IgG2a ratio in mouse serum after secondary and tertiary immunization with BoNTA/M4, BoNT/AHc. Data are presented as mean ± SEM (n = 3). * *p* < 0.05; ** *p* < 0.01; **** *p* < 0.0001; ns, not significant (*p* > 0.05).

To better understand the immune response characteristics triggered by BoNTA/M4 protein in mice, we used ELISA to detect the levels of specific IgG1, IgG2a, IgG2b, IgG3, and IgM antibodies in their immune sera following immunization. As shown in Figure 3, both BoNTA/M4 and BoNTA/Hc proteins mainly induced the production of IgG1 and IgG2a antibodies (Figure 3A,B), with minimal levels of IgG2b, IgG3,and IgM antibodies. Notably, the ratio of IgG1/IgG2a was greater than 1 (Figure 3E). The experimental results suggest that during the immune process, the BoNTA/M4 and BoNTA/Hc antigens are more inclined to activate the Th2-type immune response pathway.

### 3.5. Evaluation of the Ability to Neutralize BoNTA Toxin of Serum of Mice Immunized with BoNTA/M4 Antigen

**Figure 4:**
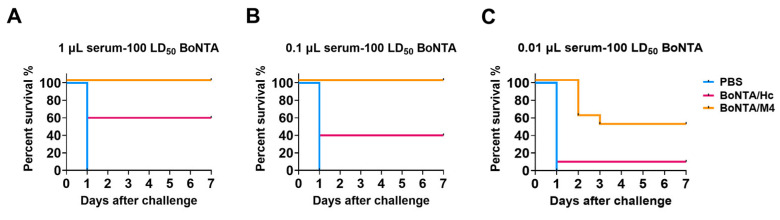
The serum of immunized mice neutralized the toxicity of 100 LD_50_ BoNTA. Three different volumes of mouse serum (1 μL, 0.1 μL, 0.01 μL) collected after immunization were mixed with BoNTA (100 LD_50_), incubated at room temperature for 30 min, and then intraperitoneally injected into mice, with 10 mice in each group. (**A**) 1 μL serum; (**B**) 0.1 μL serum; (**C**) 0.01 μL serum.

To evaluate the efficacy of BoNTA/M4 antigen immune serum in neutralizing BoNTA toxins, we mixed different volumes of serum (1 μL, 0.1 μL, and 0.01 μL) with 100 LD_50_ BoNTA and then injected intraperitoneally into mice after incubation at room temperature for 1 h. The survival status of mice was observed continuously for 7 days, and the results were compared with the positive control BoNTA/Hc. In the experiment with 1 μL serum, all mice in the PBS immunization group died, indicating that there was no protective effect of neutralizing antibodies; conversely, all mice in the BoNTA/M4 immunization group survived and showed complete protective effects while the survival rate in the positive control BoNTA/Hc group was only 60%, indicating that BoNTA/M4 immune serum showed higher efficiency in neutralizing BoNTA toxins (Figure 4A). When the serum volume decreased to 0.1 μL, all mice in the BoNTA/M4 immunization group survived, while the survival rate of mice in the BoNTA/Hc group decreased to 40% (Figure 4B). Further decreasing the serum volume to 0.01 μL resulted in a 60% survival rate in the BoNTA/M4 immunization group, compared to only 10% survival in the BoNTA/Hc group (Figure 4C). At serum volumes ranging from 1 μL to 0.01 μL, BoNTA/M4 immune serum consistently demonstrated a higher ability to neutralize BoNTA toxins compared to the positive control BoNTA/Hc.

### 3.6. Mouse Potency Bioassays

**Figure 5:**
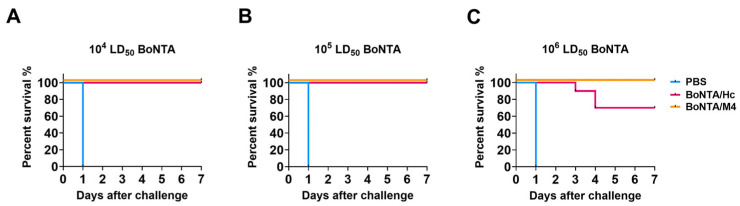
The protective effect of BoNTA/M4 and BoNTA/Hc subunit vaccines on a lethal dose of BoNTA challenge in mice. The vaccine was prepared by mixing BoNTA/M4, BoNT/A/Hc, PBS and aluminum hydroxide gel adjuvant. Mice were immunized via intraperitoneal injection every 14 days, three times in total. After 14 days of three immunizations, mice were challenged with 10^4^ LD_50_, 10^5^ LD_50_, 10^6^ LD_50_ doses of BoNTA. (**A**) 10^4^ LD_50_ BoNTA; (**B**) 10^5^ LD_50_ BoNTA; (**C**) 10^6^ LD_50_ BoNTA.

To evaluate the protective effect of recombinant BoNTA/M4 antigen against BoNTA challenge, mice were injected intraperitoneally with BoNTA at doses of 10^4^ LD_50_, 10^5^ LD_50_, and 10^6^ LD_50_ after three immunizations for 14 days. As shown in the figure, under the challenge of a 10^4^ LD_50_ dose of BoNTA, all mice in the negative control PBS group died, while all mice in the BoNTA/M4 group and positive control BoNTA/H_C_ group survived, demonstrating a complete protective effect (Figure 5A). When the challenge dose was increased to 10^5^ LD_50_, the results remained similar. All mice in the PBS group died, while all mice in the BoNTA/M4 group and BoNTA/H_C_ group survived, further confirming the complete protective ability of these two antigens at lower doses (Figure 5B). However, under the highest dose of the 10^6^ LD_50_ BoNTA challenge, there were significant differences in survival rates among the groups. All mice in the PBS group died while the survival rate of mice in the BoNTA/M4 group remained at 100%, but the survival rate of mice in the BoNT/A/H_C_ group decreased to 70% (Figure 5C). This result indicates that, under higher doses of BoNT/A challenge, the immune protection effect of the BoNTA/M4 group is significantly better than that of the BoNTA/H_C_ group.

## 4. Discussion

Since the 1940s, public attention has focused on new treatment and prevention strategies for BoNT poisoning, including nucleic acid vaccines, peptide vaccines, and subunit vaccines; however, at present there are no FDA-approved vaccines on the market [16,30]. In this study, we prepared a novel recombinant full-length detoxified protein, BoNTA/M4, by introducing four-amino-acid mutations (E224A, E262A, R363A, Y366F) to the toxicity sites of BoNTA. The result of a mouse in vivo toxicity assay shows that its toxicity is one millionth that of natural BoNTA proteins. Three immunizations with this antigen can induce high titers of neutralizing antibodies in mice, effectively protecting them from challenge with up to 10⁶ LD_50_ doses of BoNTA, and the protective effect is significantly higher than that of the BoNTA/H_C_ antigen, which is consistent with the antibody titer and neutralizing activity of the immune serum. These results indicate that BoNTA/M4, as a vaccine candidate for preventing BoNTA poisoning, can elicit a robust immune response and demonstrates good immune protection efficacy.

A core challenge in designing a full-length detoxified vaccine for BoNTA lies in maintaining its immunogenicity while eliminating its toxicity to ensure vaccine safety. Drawing on existing research on BoNTA toxicity-related sites, we performed a combinatorial mutagenesis on three types of sites within the LC, namely those involved in substrate binding, catalysis, and transition state stability. Through a crystal structure analysis of the complex formed between BoNTA and SNAP25, we aimed to further reduce toxicity and ensure that the safe dose of the candidate vaccine is much higher than the immune dose. Contrary to our expectations, BoNTA/M4, which contains four mutation sites, exhibited significantly lower toxicity than BoNTA/M6, which has six mutation sites. This suggests that certain combinations of key site mutations may not produce the anticipated effects in specific scenarios and could potentially have adverse consequences. These findings indicate that we must not only focus on the mutation effect of individual sites but must also comprehensively evaluate the synergy between mutation sites and the overall structural stability of BoNTA. Our research delves into the superposition effect of key mutation sites in BoNTA, offering valuable insights for the future development and optimization of other toxin mutants.

The FRET method failed to detect the endopeptidase activity of BoNTA/M4, but toxicity experiments showed that BoNTA/M4 still had low residual toxicity. On the one hand, this indicates that there is not an absolute correspondence between enzyme activity and toxicity, and on the other hand, it also suggests that there is still room for further improvement regarding the toxicity of BoNTA/M4. In summary, this study indicates that BoNTA/M4 has good safety and excellent immunogenicity, laying the foundation for the development of a new BoNTA vaccine.

## 5. Conclusions

In this study, we successfully engineered three detoxified, full-length toxin mutants: BoNTA/M2, BoNTA/M4, and BoNTA/M6. By assessing their median lethal doses (LD50) in mice, we found that BoNTA/M4 exhibited the most optimal safety profile. When compared to the receptor-binding domain protein BoNTA/Hc, BoNTA/M4 was able to induce higher levels of specific antibodies. Furthermore, the immune serum generated from BoNTA/M4 demonstrated robust neutralizing activity and protective efficacy against BoNTA challenges. This research not only laid a solid foundation for the development of a BoNTA vaccine but also underscored the significant potential of BoNTA/M4 as a novel vaccine candidate.

## Figures and Tables

**Table 1:** Primers used in this study.

Primer	Sequence (5′-3′)
E224A-F	CATTAGCACATGCACTTATCCATGCTGGACATCG
E224A-R	CATGGATAAGTGCATGTGCTAATGTTACTGCTGGATC
E262A-F	AGCTTTGAGGCACTTCGCACATTTGGGGGAC
E262A-R	ATGTGCGAAGTGCCTCAAAGCTGACTTCTAACCCAC
R363AY366F-F	CCTTAACGCAAAAACATTCTTGAATTTTGATAAAGCCGTATTTAAGATC
R363AY366F-R	CAAAATTCAAGAATGTTTTTGCGTTAAGGACTTTAAAAAACTTAACAAAATTATCC
H223AH227A-F	CTAAATCATTAGATAAAGGATACGCAAAGGCACTGAACGATTTG
H223AH227A-R	GCAGCGATAAGTGCAGCTGCTAATGTTACTGCTGGATCTG

The primers used in this study were synthesized by Qingke Biotechnology Co., Ltd. (Beijing, China).

**Table 2:** The medium lethal dose for mice (MLD_50_). Mice were intraperitoneally injected with corresponding doses of BoNTA/M2, BoNTA/M4, and BoNTA/M6 proteins and observed for 7 days.

Mutant Protein	Challenge Dosage (ng)	Survial Percent	1 LD_50_ (ng)
BoNTA/M2	1000	0%	136.77
	500	0%	
	200	50%	
	100	100%	
	50	100%	
BoNTA/M4	10,000	0%	5000
	5000	50%	
	2000	100%	
	1000	100%	
	500	100%	
BoNTA/M6	5000	0%	674
	2000	0%	
	1000	25%	
	500	50%	
	200	100%	

## Data Availability

The data presented in this study are available on request from the corresponding author.
